# Reducing the Emotionality of Auditory Hallucination Memories in Patients Suffering From Auditory Hallucinations

**DOI:** 10.3389/fpsyt.2019.00637

**Published:** 2019-09-18

**Authors:** Suzy Johanna Martina Adriana Matthijssen, Ivo Heitland, Liselotte C. M. Verhoeven, Marcel A. van den Hout

**Affiliations:** ^1^Altrecht Academic Anxiety Centre, Altrecht GGZ, Utrecht, Netherlands; ^2^Clinical Psychology, Utrecht University, Utrecht, Netherlands; ^3^Department of Psychiatry, Social Psychiatry and Psychotherapy, Hannover Medical School, Hanover, Germany; ^4^Personality Disorders, GGZ Centraal, Amersfoort, Netherlands; ^5^Hospital psychiatry, mood disorders and anxiety, Meander Medical Centre, Amersfoort, Netherlands

**Keywords:** eye movement desensitization and reprocessing, psychosis, working memory taxation, modality specific taxation, auditory emotional memories, auditory hallucinations

## Abstract

Eye movement desensitization and reprocessing (EMDR) therapy targets emotionally disturbing visual memories of traumatic life events, and may be deployed as an efficacious treatment for posttraumatic stress disorder. A key element of EMDR therapy is recalling an emotionally disturbing visual memory while simultaneously performing a dual task. Previous studies have shown that auditory emotional memories may also become less emotional as a consequence of dual tasking. This is potentially beneficial for psychotic patients suffering from disturbing emotional auditory memories of auditory hallucinations. The present study examined whether and to what extent emotionality of auditory hallucination memories could be reduced by dual tasking. The study also assessed whether a modality matching dual task (recall + auditory taxation) could be more effective than a cross modal dual task (recall + visual taxation). Thirty-six patients suffering from auditory hallucinations were asked to recall an emotionally disturbing auditory memory related to an auditory hallucination, to rate emotionality of the memory, and to recall it under three conditions: two active conditions, i.e., visual taxation (making eye-movements) or auditory taxation (counting aloud), and one control condition (staring at a non-moving dot) counterbalanced in order. Patients re-rated emotionality of the memory after each condition. Results show the memory emotionality of auditory hallucinations was reduced and the active conditions showed stronger effects than the control condition. No modality-specific effect was found: the active conditions had an equal effect.

## Introduction

Although auditory hallucinations (AHs) are associated foremost with schizophrenia spectrum disorders (approximately 70% of the people with a diagnosis of schizophrenia report AH) (1), they are also documented in patients with posttraumatic stress disorder (PTSD; 40%–50%), bipolar I disorder (37%), borderline personality disorder (30%), major depression (10%–23%), obsessive–compulsive disorder (14%), dementia (14%), and Parkinson disease (10%) (2). AHs are also common in the general population. A meta-analysis by Maijer et al. (3) reported a mean lifetime prevalence for AHs in the general population of 9.6%.

### Therapies for AHs

AHs can cause great distress and impairment in daily life and warrant effective treatment strategies. Research on treatment strategies most commonly focusses on auditory *verbal* hallucinations (AVHs) or hearing voices. Sometimes the definitions AVHs and AHs are interchanged, although AHs also include *non-verbal* hallucinations, whereas AVHs do not. Since AHs and AVHs occur mostly in patients with schizophrenia (4, 5), treatment strategies are derived mainly from studies on these patients. The treatment of choice for AHs is antipsychotic medication, which induces a rapid decrease in hallucination severity (6). Most patients with a first psychotic episode show a decline in psychiatric complaints in response to the medication (7). On the other hand, in approximately 25 to 30% of the patients, antipsychotic medication is ineffective in the treatment of AHs (8, 9). In addition, antipsychotic medication has several significant side effects (8). Furthermore, a substantial proportion of patients do not adhere to drug treatment (9). Lacro et al. (10) found a mean rate of nonadherence ranging from 41.2% to 49.5%, depending on the criteria used for non-adherence. Therefore, despite the large contribution of antipsychotic medication in the treatment of AHs (and AVHs), development of novel or enhancement of existing treatment strategies is important.

Treatment strategies for AHs can be aimed at reducing the *frequency* and duration of hallucinations, but can also be aimed at a *reduction of the distress caused by hallucinations*. Cognitive behavioral therapy (CBT) aims at this reduction of distress by focusing on the management of AHs. It is recommended in the National Institute for Health and Care Excellence guidelines (11) in addition to medication for the treatment of hallucinations in adult patients with psychoses and schizophrenia. In a meta-analysis, van der Gaag et al. (12) reported small to medium effect sizes (*Hedges’ g* = .31 to .49) for CBT on hallucination total scores of AHs in patients with AHs. As hallucination total scores were calculated, no differentiation of the effect on different symptom aspects of AHs (e.g., burden, frequency, and loudness) was possible. A review by Mazmanian et al. (13) also discussed CBT effects in schizophrenia patients with AHs. They concluded that CBT had a beneficial effect (both in individual and in group therapy) on different clinical parameters (e.g., stress generated by AHs, obedience to the voices, intensity of the voices, and social skills). Unfortunately, the authors did not report effect sizes.

Other non-medication treatment strategies for AHs with different strengths of evidence are transcranial magnetic stimulation (14), AVATAR therapy (15), Acceptance and Commitment Therapy (16), mindfulness (17), relating therapy (18), and voice dialogue [e.g., Ref. (19)].

### Eye Movement Desensitization and Reprocessing

Eye movement desensitization and reprocessing (EMDR) therapy is an evidence-based therapy for posttraumatic stress disorder [PTSD; e.g., Refs. (20–24)]. One of the core symptoms of PTSD is the repeated re-experiencing of traumatic events by intrusions from memory (25). EMDR therapy aims at reducing the emotionality of these intrusive memories and thereby reducing PTSD symptoms. The key element of the therapy is letting the patient simultaneously recall an emotionally disturbing memory and move their eyes back and forth horizontally. One explanation of how EMDR yields its effects is provided by the working memory (WM) taxation hypothesis. WM has limited capacity (26). Holding an emotional memory in mind being one task, and making eye movements (EM) another, forces competition in performing the two tasks, as they both tax the limited capacity of WM (27–29). By recalling a memory, it becomes unstable, and in this transient state, memories are susceptible to the incorporation of new information (30). As a consequence of the competition, the unstable memory becomes less emotional during recall and the less emotional memory is stored back in long-term memory. This result has been found in a series of experiments, which showed that a period of recall + EM not only blurred memory during recall, but also during future recalls, when no EMs were made (23, 29). In line with the WM taxation hypothesis, there is evidence that not only EM but also any dual task that sufficiently taxes WM during memory recall elicits this effect (28, 31–34). Typically, in EMDR, visual memories are targeted, yet analogue studies have also shown autobiographical emotional memories with a predominant auditory component, which are mainly experienced as auditory, can be targeted (33, 35, 36). A study by Matthijssen et al. (37) has also shown that auditory memories can be made less emotional in PTSD patients.

There is a large overlap in symptomatology of auditory intrusions in PTSD and AHs in psychotic disorders. Morrison et al. (38) report that “Flashbacks” or intrusive recollections often appear to take the form of auditory, visual, tactile, and/or olfactory hallucinations. McCarthy-Jones and Longden (39) report that the content of AVHs relates to earlier traumatic events in a similar way in PTSD and schizophrenia. Considering the overlap in symptomatology between AHs and auditory intrusions in PTSD and the assumed relation of AVHs to earlier trauma EMDR therapy could be considered for alleviating symptoms associated with AHs. However, providing trauma treatment to patients with a psychotic disorder is uncommon and these patients are often even excluded from PTSD treatment studies (40). Van den Berg et al. (41) studied the treatment of PTSD in subjects with a schizophrenia spectrum disorder using EMDR and prolonged exposure. Both interventions were more effective than a waiting list condition and not only reduced PTSD symptoms, but also paranoid thoughts. Even so, more patients remitted from schizophrenia, as measured with a Structured Clinical Interview for Symptoms of Remission (42, 43) for the Positive and Negative Syndrome Scale (SCI-SR-PANSS) (44, 45). However, the severity of AVHs remained unchanged (45).

### Modality-Specific Dual Tasking

In clinical practice, EMDR is typically performed by allowing the patient retrieve an emotionally disturbing visual memory and simultaneously make horizontal EM. In these cases, there is a *match* between the visual modality of the intervention (EM) and the *visual* modality of the recalled memory. However, targeting disturbing auditory memories of AHs with EM would result in a *mismatch* between the modality of the intervention (visual) and the modality of the recalled memory (auditory). The WM comprises a central executive system (CES), which is engaged when attention needs to be divided, and two slave systems where modality-specific information is processed: the visuospatial sketchpad (VSSP) responsible for processing visual and spatial information and the phonological loop (PL), which is responsible for auditory and verbal processing (26, 46). The VSSP is thus involved in visual and the PL in auditory imagery (35). Some studies explored the effects of modality matching dual tasking and cross modality taxing in autobiographical memories. Kemps and Tiggemann (33) conducted a study in which undergraduates were instructed to recall a specific visual or auditory image of happy and distressing memories, while they were exposed to three different conditions [EM, articulatory suppression, and a control condition (CC)]. Concurrent articulation reduced vividness and emotional intensity ratings of auditory images to a greater extent than did EM, whereas concurrent EM reduced ratings of visual images more than articulatory suppression. Matthijssen et al. (36) asked undergraduates to recall an auditory and visual emotionally disturbing memory and to perform a random interval repetition task. The results showed that modality-matching dual-tasking was more taxing during memory recall, resulting in larger reaction time delays than cross-modal taxing. Kristjánsdóttir and Lee (35) found quite different results. They asked participants to recall an unpleasant autobiographical memory while performing EM, listening to counting or a CC (short exposure). They found that EM led to a greater decrease in vividness than listening to counting and that EM and listening to counting were equally effective in reducing emotionality. The effects were found irrespective of the modality of the memory, which led them to conclude that there was no modality-specific benefit in taxing. Matthijssen et al. (37) found similar results. They asked PTSD patients to recall an auditory and visual intrusive memory under three alternating conditions (EM, counting, staring at a non-moving dot). Auditory memories decreased in emotionality in all conditions, and no modality-specific benefit was found.

In summary, from a general WM approach and earlier findings (28, 35, 37, 47–49), one can assume that taxing WM during memory recall would result in larger decreases in emotionality scores than no dual taxing. Results on a modality-specific benefit however are less clear. Some studies suggest that competing tasks that are matched with the modality of the memory result in a greater reduction of vividness and emotional intensity ratings compared with tasks that are not matched (33, 36), but other studies fail to find this modality-specific benefit (35, 37).

To the best of our knowledge, no studies have tested whether EMDR targeting emotionally disturbing *auditory memories* of *AHs* is effective in reducing emotionality of the hallucination memories or maybe even the emotionality of the hallucinations themselves. The aim of the present study therefore is to assess if emotional intensity of *an AH memory* in patients suffering from AHs can be reduced with dual tasking, a crucial ingredient of EMDR. It is hypothesized that dual tasking makes AH memories less aversive. The second aim of the present study is to test whether a modality matched (auditory) task is more effective than a cross modal (visual) task in reducing the emotionality of disturbing auditory memories of AHs. One could suspect a modality-specific benefit causing larger decreases in emotionality than cross modal taxing. On the other hand, the benefit of modality-specific taxing was not replicated in all studies.

## Methods

### Patients

To obtain sufficient power for frequentist analyses (power 0.8, with an α-level of 0.05 and an expected medium effect size, f = 0.25), 36 patients had to be included. For the study, 38 patients suffering from AHs were recruited. Two patients were excluded. One patient had insufficient mastery of the Dutch language. The other patient expressed psychotic symptoms that interfered with the experiment. In total, data from 36 patients (23 males, 13 females) with a mean age of 39.56 (*SD* = 11.45, range 18–64) were collected. Thirty-five patients were recruited at several Faculty Assertive Community Treatment Centers of GGZ Centraal, and one patient at the Meander Medical Center, the Netherlands. All patients received treatment as usual, which mainly consisted of antipsychotic medication and/or psychological treatment and/or supportive counseling by psychologists, caseworkers, or psychiatrists. Although data from 36 patients were collected, data from only 33 patients (20 males, 13 females) with a mean age of 40.03 (*SD* = 11.63, range 18–64) were included into the analysis. The reason to exclude three patients was that although intended to present all conditions [visual taxation (VT), auditory taxation (AT), and CC] twice, these patients did not complete all conditions at least once. The patients reached a Subjective Units of Disturbance (SUD) score of zero, indicating no emotional disturbance when recalling the memory, before all conditions (VT, AT, CC) were presented once. Out of the 33 patients, 87.9% was classified with a psychotic disorder based on DSM-IV-TR criteria (50). For specific patient characteristics, see **Table 1**. Inclusion criteria for the study were that the patient had to suffer from AHs, had an estimated IQ ≥ 80, was ≥18 years of age, and was able to recall an emotionally disturbing auditory memory of a predominantly AH, which had to be rated at least 50% auditory in content. The AH could be verbal or non-verbal. The aversiveness of the experienced memories is an analogue of the aversiveness of PTSD memories, not the content per se (verbal or non-verbal). Exclusion criteria were a high acute suicide risk, insufficient mastery of the Dutch language, and severe visual or hearing impairment(s). IQ, suicide risk, and mastery of the Dutch language were estimated by the therapist who referred the patient for the study. IQ was rated on the basis on school or work performance and/or clinical impression of the referring therapist. IQ was not assessed with a validated instrument. Axis I or axis II diagnoses were clinician-based and made by psychiatrists and mental health or clinical psychologists, who are eligible and trained to establish diagnoses.

**Table 1:** Patient characteristics (*N* = 33).

Characteristics	*N* (%)
Gender	
Female	13 (39.4%)
Male	20 (60.6%)
Axis I disorder	
Psychotic disorder	16 (48.5%)
Psychotic disorder + addiction disorder (+ADHD)	9 (27.3%)
Psychotic disorder + other diagnoses	4 (12.2%)
Mood disorder with psychotic features (+ other)	3 (9.1%)
Anxiety disorder + PTSD + autism	1 (3%)
Comorbid axis II disorder	
No diagnosis	28 (84.8%)
≥ Axis II diagnosis	5 (15.2%)
Education level	
Not finished any school	1 (3%)
Primary school	9 (27.3%)
Secondary school	11 (33.3%)
Lower vocational education	5 (15.2%)
Secondary vocational education	4 (12.1%)
Higher professional education	2 (6.1%)
University	1 (3%)
Psychopharmacological drugs	
No use of medication	1 (3%)
Antipsychotics (AP)	8 (24.2%)
Antipsychotics (AP) + Benzodiazepines (BD)	10 (30.3%)
Antipsychotics (AP) + Antidepressants (AD)	2 (6.1%)
AP + BD + AD	2 (6.1%)
AP + Other (single or combination)	9 (27.2%)
AD + BD	1 (3%)

The mean age of onset of voice-hearing was 22 years (*SD* = 10.24). Patients reported hearing between 0 and 1000 voices last week, most commonly hearing three voices (30.3%, *N* = 10). For specific characteristics of the AHs, see **Tables 2** and **3**.

**Table 2:** Characteristics of AHs last week measured with the PSYRATS-AH (*N* = 33) (PSYRATS-AH, Psychotic Symptoms Rating Scale - Auditory Hallucinations).

	N (%)
**Modalities**	
No other modalities	10 (30.3%)
Visual hallucinations	5 (15.2%)
Olfactory hallucinations	1 (3%)
Tactile hallucinations	2 (6.1%)
Multiple modalities	15 (45.5%)
**Frequency**		
No voices present or less than once per week	1 (3%)
At least once a week	3 (9.1%)
At least once a day	4 (12.1%)
At least once per hour	8 (24.2%)
Continuously or almost continuously	17 (51.5%)
**Duration**		
No voices	1 (3%)
A few seconds	6 (18.2%)
Several minutes	7 (21.2%)
At least 1 h	4 (12.1%)
Several hours	15 (45.5%)
**Location**		
No voices	1 (3%)
Only voicwes in the head	7 (21.2%)
Voices close to the ears or head (and possibly inside)	10 (30.3%)
Voices in or close to the ears and further away	4 (12.1%)
Voices from the surrounding, further away from the head	11 (33.3%)
**Loudness (last time heard)**	
No voices	1 (3%)
Quieter than own voice, whispering	13 (39.4%)
As loud as own voice	12 (36.4%)
Louder than own voice	3 (9.1%)
Very loud, shouting	4 (12.1%)
**Attribution (at moment of interview)**	
No voices	–
Convinced voices are internally generated and connected with the patient himself	7 (21.2%)
Less than50% convinced voices are caused by external cause	5 (15.2%)
50, but less than 100% convinced voices have external cause	11 (33.3%)
100% sure voices are externally caused	10 (30.3%)
**Negative content**	
No negative content	3 (9.1%)
Now and then negative content	5 (15.2%)
Less than 50% unpleasant	4 (12.1%)
More than 50% unpleasant	11 (33.3%)
All content is unpleasant	10 (30.3%)
**Severity of negative content**	
Not unpleasant or negative	–
Certain amount of negative content but not aimed at the person or his family	–
Negative content aimed at the behavior of the person	1 (3%)
Negative content aimed at the self-concept of the person	20 (60.6%)
Threats aimed at the person or its family	12 (36.4%)
**Burden**	
Voices are never unpleasant or annoying	2 (6.1%)
Sometimes annoying, but mostly not	3 (9.1%)
Equally annoying as not annoying	3 (9.1%)
Majority of the voices unpleasant or annoying	9 (27.3%)
Voices are always unpleasant or annoying	16 (48.5%)
**Intensity of the burden**		
Voices cause no discomfort	2 (6.1%)
Voices cause limited discomfort	4 (12.1%)
Voices cause moderate discomfort	10 (30.3%)
Voices cause severe discomfort	10 (30.3%)
Voices cause extreme discomfort	7 (21.2%)
**Disturbance in everyday life**	
No disturbance of everyday life	–
Limited disturbance (e.g., concentration)	3 (9.1%)
Moderate disturbance (e.g., hindering daily activities)	15 (45.5%)
Severe disturbance (e.g., often requiring hospitalization)	15 (45.5%)
Total disturbance of life (e.g., constant hospitalization required)	–
**Experienced control over voices**	
Control	1 (3%)
Mostly some control	6 (18.2%)
Half of the time some control	1 (3%)
Mostly no control	11 (33.3%)
No control	14 (42.4%)

**Table 3:** Patients’ beliefs, emotions, and behavior about their AHs measured with the VOS-R (N = 33) (VOS-R, Vragenlijst Opvattingen over Stemmen-Revised; in English, BAVQ-R, beliefs about Voices Questionnaire-revised).

Characteristics	M (*SD*)
Malevolence (0–18)	11.58 (4.21)
Benevolence (0–18)	2.88 (3.61)
Power (0–18)	10.58 (4.15)
Involvement (0–24)	4.06 (5.04)
Resistance (0–27)	18.52 (5.26)

### Procedure

Study procedures were approved by the Medical Research Ethics Committee of the University Medical Center Utrecht (The Netherlands) (NL54140.041.15, protocol number 15/428D). The study was conducted by two trained EMDR therapists. To enhance clinical relevance, the study was designed with EMDR features that mimic clinical practice as much as possible (e.g., session in the therapy room and adhering as much as possible to the EMDR protocol). Therapists from participating mental healthcare institutions were asked to screen their caseload for eligible patients. Patients were provided with an information letter and were able to consider participating for at least a few days. Patients who gave oral consent to their therapist were referred to the researcher. Because patients gave oral consent or refused participation to their therapist, the researchers are unaware how many patients refused participation.

**Figure 1:**
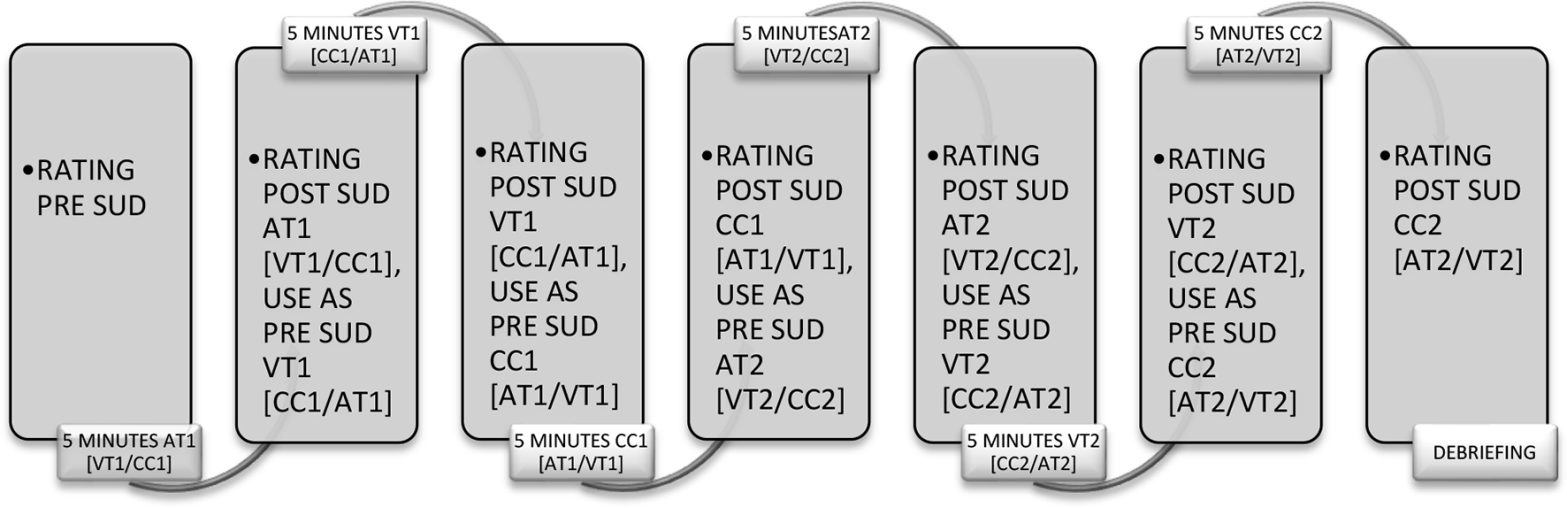
Timeline of the procedure (SUD, Subjective Units of DisturbanceUD scores; VT, Visual Taxation; AT, Auditory Taxation; CC, Control Condition).

After providing written informed consent, patients completed two questionnaires concerning information about the characteristics of their AHs, the Psychotic Symptoms Rating Scale–Auditory Hallucinations (PSYRATS-AH), and the Beliefs about Voices Questionnaire-revised [BAVQ-R; in Dutch, Vragenlijst Opvattingen over Stemmen-Revised (VOS-R)] (see Materials for further elaboration on the questionnaires). Patients were then instructed to recall an emotionally disturbing auditory memory of an AH and indicate to what degree the content was auditory by indicating this on a 100 mm Visual Analogue Scale (VAS) ranging from 0 (content of the memory is not auditory at all) to 100 (content of the memory is completely auditory). The memory had to be rated at least 50% auditory in content to be included. If memories were rated below 50% auditory in content, patients were asked to recall another auditory memory. Furthermore, other sensory modalities (visual, gustatory, kinesthetic, and olfactory) were determined not to be more dominant in the selected memory than the auditory modality. Patients were asked to point out the presence of other modalities in the memory by dividing a 100 mm VAS between the different modalities present in the memory. Then, patients were asked to recall the memory and to rate how disturbing the memory was when being recalled on a scale from 0 (no disturbance) to 10 (maximum disturbance). After this, patients were instructed to recall the auditory memory again and to either consequently make horizontal EMs at a speed of one cycle eye movements per second (VT), to count down aloud from 1000 (AT), or to stare at a non-moving dot (CC). The study used step one, two, and three (introduction, assessment, and desensitization) from the standard Dutch EMDR protocol (51). The protocol was slightly altered for this study to fit the auditory content and the research set-up. All words referring to visual images were changed to refer to auditory content. Every condition (AT, VT, and CC) was offered five times for periods of 1 min each before switching to the next condition. Each condition was offered twice, resulting in recalling the memory approximately 30 min in total. After each 1 min period, the condition was interrupted and the patient was asked what arose to mind. The answer was, regardless of the content, followed by the researchers’ instruction “concentrate on that” and followed by the next 1-min period. Before alternating to another condition, the SUD was re-rated (by verbal expression of the patient) on a scale from 0 to 10. The experiment continued until all conditions were offered twice or until the patient reached SUD score of zero. For an outline of the procedure, see **Figure 1**.

During all conditions, participants were seated in front of a light bar. During the VT condition, a moving dot was displayed. During the AT condition, the bar displayed nothing, and during the CC, the bar displayed a non-moving dot in the center of the bar. The speed used for the moving dot in the VT condition and the type of counting task were copied from studies of Engelhard et al. (31) and van den Hout et al. (34, 52). In these studies, individuals carried out a simple reaction time task, where they had to press a button every time a stimulus was perceived. An increase in response time was observed when an additional task was given. This resulted in a quantitative index of the degree to which the additional task was cognitively taxing. In these studies, the delay in response time as a result of EM with 1 cycle per second (RT of 115 ms) and the response delay as a result of a 1000-to-1 countdown (RT of 97 ms) were comparable. These two tasks were therefore considered as equally taxing the WM.

### Design

The study had a 2 (Time: pre vs. post) × 3 (Condition: VT, AT and CC) repeated measures within-subject design, with SUD scores as a dependent variable. Data from patients, who received all conditions at least once, were included in the analyses, resulting in including data from 33 patients. For many of the participants who did not reach a SUD score of zero after all conditions were offered once (i.e., “the first round”), the SUD scores were substantially reduced at the end of the first round, which limited room for (differential) effect during the second round. Only in 22 patients all conditions were presented twice. This was a clinically very welcome and encouraging result, demonstrating reduction in SUD scores. However, this resulted in discarding part of the data and including only data obtained in “the first round” in the analyses.

### Materials

**PSYRATS-AH.** (53) A semi-structured interview with a five-point ordinal scale that measures the severity of different dimensions of AHs. The AH scale consists of 11 items and has been found to have excellent inter-rater reliability and good validity and is sensitive to change (53).
**BAVQ-R.** ([54], Dutch version: VOS-R). A 35-item self-report questionnaire with a four-point ordinal scale with five sub-scales, three of which focus on a person’s beliefs about the dominant voice (omnipotence, malevolence, and benevolence) and two scales that focus on emotional and behavioral responses (resistance and engagement). The subscales have good internal reliability, with correlations between malevolence and resistance, and benevolence and engagement suggesting construct validity (54).
**Subjective Units of Disturbance (SUD).** The SUD score is a self-assessment score with a scale that assesses the subjective intensity of disturbance or distress felt by the patient at the moment of recalling the auditory memory of the AH and ranges from 0 (no disturbance) to 10 (maximal disturbance). The effect of each condition was measured by the SUD-difference score, which was calculated by subtracting the SUD score of the memory post-condition from the SUD scores pre-condition.

### Data Analyses

Data were analyzed using Bayesian statistics^1^. Bayesian approach provides relative support for a pre-specified model or models (55) instead of depending on dichotomous decisions as provided by p-value significance testing. It enables direct testing of theoretical expectations without the need for *post hoc* pairwise comparisons. Furthermore, one can test different models at once. The results are expressed in terms of Bayes factors (BFs), which represent the level of evidence for one model compared to a model without constraints or against its complement (another pre-specified model). A BF value greater than 1 indicates that the data support the model, and the higher this factor, the more support. A BF value less than 1 indicates no support for the model. For further reading on Bayesian analyses, see Wetzels et al. (56) and Krypotos et al. (57). Data were analyzed using the software package BIEMS (Bayesian inequality and equality constrained model selection). BIEMS was developed for computing BFs between hypotheses with order constraints and/or equality constraints between means and regression parameters in multivariate normal linear models (see 58, 59). Four pre-specified models were formulated to asses the effect of the conditions (Model 1, 2, 3, 4):

- Model 1: AT (pre-post) = VT (pre-post) > CC (pre-post). There is a general taxation effect but no modality-specific effect, resulting in SUD decreases in the AT and VT conditions, but not in the CC or larger SUD decreases in the AT and VT conditions compared to the CC.
- Model 2: AT (pre-post) > VT (pre-post) > CC (pre-post). There is a modality-specific effect and a general taxation effect. This results in larger SUD decreases in the AT and VT conditions than the CC, and a superimposed effect of the AT condition over the VT condition.
- Model 3: AT (pre-post) > VT (pre-post) = CC (pre-post). There is only or mostly a modality-specific effect. This results in larger SUD decreases in the AT, but no decrease or smaller decreases in the VT condition and the CC.
- Model 4: AT (pre-post) = VT (pre-post) = CC (pre-post). There is no effect of general taxation and no effect of modality specificity. This results in the same SUD difference scores in all conditions.

## Results

**Figure 2:**
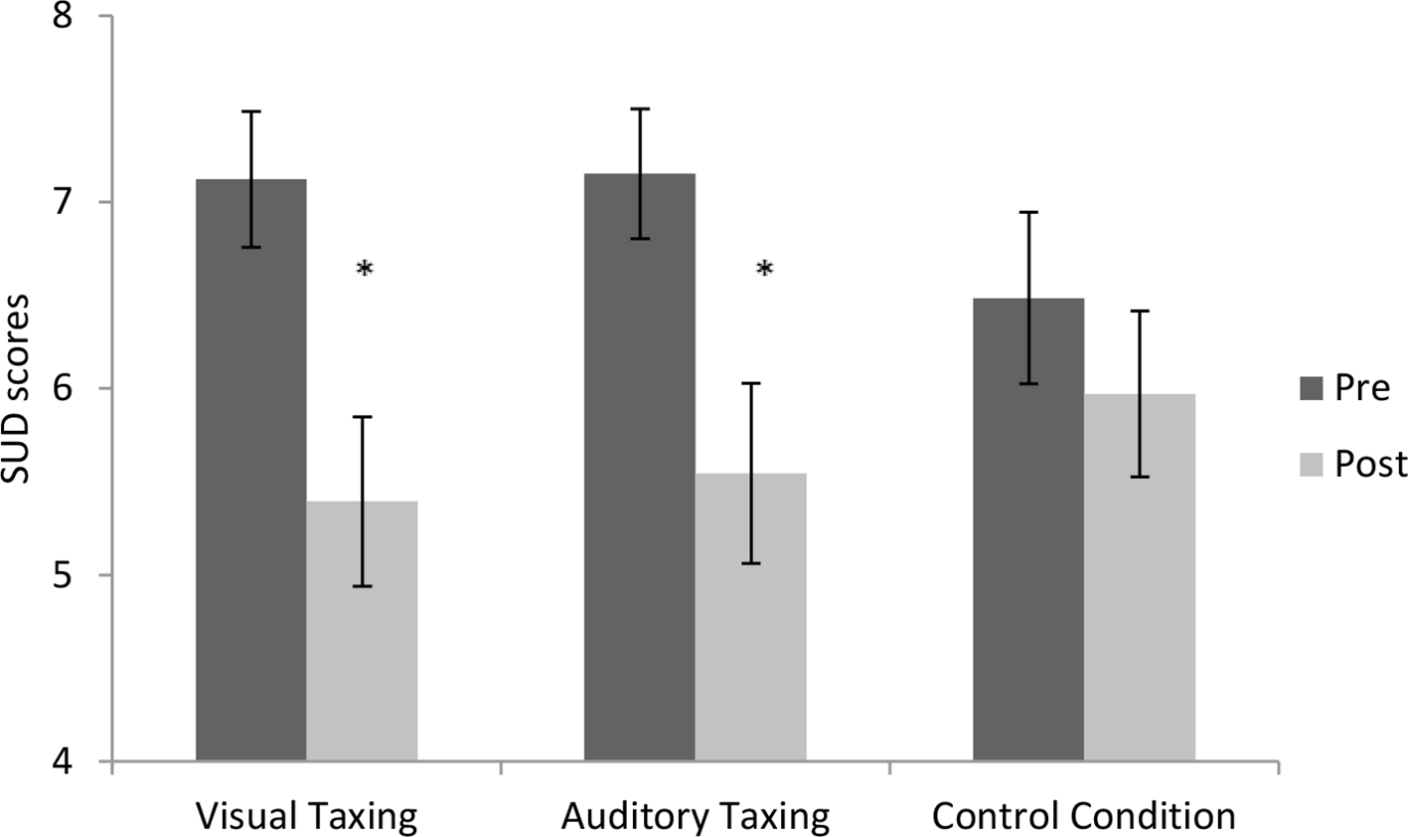
Pre- and post SUD scores per condition. Error bars depict +-1 S.E.M. (* = p < .05) (SUD, Subjective Units of Disturbance).

Findings are graphically represented in **Figure 2**.

### General WM Taxation

To investigate if the emotional intensity of a memory of an AH could be reduced by WM taxation, models 1, 2, and 4 were taken into account. Support for model 1 would indicate that the active conditions (AT and VT) would show larger decreases from pre to post than the CC, indicating support for a general WM taxation effect. Support for model 2 would indicate a general WM taxation effect and modality-specific effect, and support for model 4 would indicate no difference of the active conditions compared to the CC. Support for WM taxation in general was found [BF_1_ = 5.8, model 1: AT (pre-post) = VT (pre-post) > CC (pre-post)]. According to Lee and Wagenmakers (60), this reflects moderate support (BF’s > 3 are categorized as moderate evidence). However, support was also found for the lack of difference between the three conditions [AT (pre-post) = VT (pre-post) = CC (pre-post); BF_4_ = 1.95], suggesting that there was neither a general nor a modality-specific effect. However, the support for this model was substantially lower and classified as anecdotal (60). Also, model 2 [AT (pre-post) > VT (pre-post) > CC (pre-post)], which takes general taxation into account besides a modality-specific effect, shows anecdotal support (BF_2_ = 2.21). (Further elaboration on this model can be found under Modality Specificity.) Analyses in which PTSD patients were compared to non-PTSD patients were not possible, since the subgroup of PTSD patients was too small. It is however very plausible that the number of patients in the sample actually suffering from PTSD is much higher, since the diagnosis is often missed in psychotic patients (61).

### Modality Specificity

For testing the effect of modality-specific taxing, two models were taken into account. Support for model 2 [AT (pre-post) > VT (pre-post) > CC (pre-post)] would indicate that dual taxing in general would result in larger decreases in emotionality and there would be an effect of modality-specific taxing resulting in larger decreases in the AT condition than the VT condition, thus implying impact of dual taxing in general and a modality-specific taxing effect. Anecdotal support (60) for the model was found (BF_2_ = 2.21). Support for model 3 [AT (pre-post) > VT (pre-post) = CC (pre-post)] would also indicate support for a modality-specific taxation effect, but less or no effect of general WM taxation by finding only or larger SUD decreases in the AT condition. Model 3 was not supported (BF_3_ = .97).

## Discussion

The first aim of the present study was to test whether emotionality of an auditory memory of an AH could be reduced by applying dual tasking during recall of the auditory memory. Patients suffering from AHs were asked to recall an emotional auditory memory of an AH under three conditions: VT (making horizontal EMs) as usually performed in EMDR, AT (counting), and a CC (staring at a non-moving dot). The second aim was to assess if a modality matching dual task (AT + recall of the auditory memory) would be more effective in reducing emotionality than a modality mismatching dual task (VT + recall of the auditory memory). A customized Dutch version of the standard EMDR protocol was used. The protocol was adapted to refer to auditory content (as opposed to the usual visual content). To the best of our knowledge, this is the first study to examine if and to what extent the emotional intensity of AH memories can be reduced by dual tasking.

Results showed that emotional intensity (measured with the SUD) of AH memories can be reduced. The model in which an equal superior effect was presumed of the auditory and visual taxing conditions in reducing emotionality compared to the CC was most supported by the data (BF_1_ = 5.8). The support is categorized as moderate support (60). This corresponds to null hypothesis significance testing (see the Appendix) were emotional intensity shows a significant decrease over time and a trend is observed when comparing both active conditions (VT and AT) to the CC (*p* = .07)

The second aim of the study was to assess whether modality-specific taxing was more effective in reducing SUD scores than cross modal taxing. Analyses show no modality-specific benefit. The AT and VT conditions both reduce emotionality. Some support was found for equal effects in all the conditions (BF_4_ = 1.95), and some support was found for superiority of AT over VT in reducing emotionality and both over the CC (BF_2_ = 2.21), but both levels of support are classified as anecdotal (60). Furthermore, outperformance of modality-specific taxation shows no support (BF_3_ = .97).

The fact that the emotionality of auditory memories could be reduced in autobiographical memories with (some) auditory content is in line with the results of previous studies with non-clinical samples (33, 35, 36) and PTSD patients (37). This present study differed from the previous studies, because it focused specifically on AH memories. Results are partly in line with the WM taxation hypothesis. Bayesian analyses show the most support for larger decreases in emotionality of the memory in the active (auditory and visual) taxation conditions, which were supposed to be more taxing than the CC, as well as in null hypothesis testing which showed a trend indicating more effect of the active conditions compared to the CC. A study by van Veen et al. (48) showed that the greater the amount of taxation during recall, the larger the decrease in vividness and emotionality of an emotionally disturbing memory. In the present study, both active conditions were supposed to evoke an equal amount of taxation on the WM, and more so than the CC, which would explain similar results in these conditions. There was, however, *moderate* support for the outperformance of the active conditions over the CC. One explanation for not finding a very clear difference of effect between the CC and the active conditions can be due to the effect of and the specifics of the control task. The control task, staring at one point on the light bar, was experienced and executed quite differently by patients. Some patients mentioned seeing the dot change in color; others tried very hard to focus on the non-moving dot. Possibly the control task also taxed WM and perhaps more so in patients suffering from AHs compared to healthy controls, since WM is often affected in patients diagnosed with schizophrenia (62). Similar effects were evoked by comparable conditions in other studies. In a study by Sack et al. (63), patients suffering from PTSD were randomly allocated to either exposure with EM, exposure with fixating on a nonmoving hand, and exposure without the explicit task of fixating on an external focus of attention (e.g., eyes closed or eyes open and looking into the room without focus). Exposure with EM and exposure with fixation on a nonmoving hand showed the same effect on reduction of PTSD symptoms. They concluded that performing EM had no advantage over fixation on a nonmoving hand and they highlighted the need for further research on the exact mechanism why an external focus of attention (moving or nonmoving hand) might help to increase treatment effects during exposure therapy. Dunn et al. (64) and Yaggie et al. (65) report similar results of these types of CCs. Stickgold (66) points out that eye fixation maintained for 30 s appears to produce a shift in mental state and “if such a state shift also facilitates trauma processing, then its use as a CC would reveal no relative benefit for bilateral movements, leading to a false rejection of their efficacy.” On the other hand, Stickgold points out that this could also be the contrary in which this shift in mental state could be detrimental to trauma processing. The correct CC therefore should be the absence of intentional EM or nonmovements (66). The type of CC used in the current study should be considered a limitation.

The lack of a modality-specific taxing effect suggests that the sheer memory taxation overshadows any effects from modality specificity. It contradicts studies where superior effects of modality-specific taxing were found (27, 33, 36, 67–69). However, it is in line with several earlier studies failing to find a modality-specific effect (35, 37). Several explanations can be given for the lack of finding a modality-specific effect. The effect of modality specificity could be absent. However, this seems highly unlikely, since a very strict experimental study (36) did find this effect and it was found in multiple studies. A second explanation is that the effect of modality-specific taxation is small. This is in line with results from Kemps and Tiggemann (33) who suggest a large general WM taxation effect and a smaller superimposed modality-specific effect. A patient sample was used in the current study, which could have led to more confounding factors than the use of undergraduates as a participant sample. Some patients were not able to follow the moving dot, which was set standard speed; some experienced difficulty in counting. Also, some patients were bothered by AHs during the intervention. Furthermore, since only 9 out of 33 patients finished primary school and 1 did not finish primary school at all, one could argue that some patients maybe were not capable to follow instructions and did not have an IQ > 80. Next, studies should include validated measurements for IQ if this is an inclusion criterion. These above mentioned variables were not taken into account, but it is not unlikely that it affected the results.

The results show that emotionality of the disturbing AH memories decreased, but the effects on the frequency, severity, or perceived distress of the AHs themselves were not taken into account. When administering EMDR therapy in the treatment of PTSD, patients are asked to recall an emotionally disturbing visual memory of the trauma and rate its emotional adversity. The visual intrusions experienced outside treatment are voluntarily activated in treatment. It seems plausible that the positive outcome of EMDR is the result of generalizing recued aversiveness of the voluntarily recalled visual memory within the session to the visual memory recalled outside the treatment context. In the present study, patients were asked to recall their most upsetting hallucination memory. Yet, how similar was the experienced voluntarily activated emotionally disturbing auditory memory of the AH within the session to real life involuntary AHs? It seems plausible that the same reduced aversiveness of the voluntarily recalled auditory memory within the session is generalizable to the auditory memory recalled outside the treatment context. Earlier studies showed that PTSD symptoms are reduced concomitantly with an EMDR-induced reduction of the emotional intensity of visual memories in patients with PTSD (20). This could suggest that emotionality reductions of the auditory memories in patients with AHs may also decrease symptoms of AHs. Though feedback from individual therapists suggested a strong effect on the hallucinations for some of the patients included in the study, no post-measurements or follow-up assessments of the PSYRATS-AH and BAVQ-R were included to objectify the effect on the AHs and this makes it impossible to determine whether treatment gain on the frequency, severity, or perceived distress of AHs was achieved. Furthermore, it is not clear whether the effects on emotionality scores are short-lived or lasting. The study, being experimental in nature, consisted of one intervention session only. Most patients experienced multiple AHs, and it was hypothesized that for most patients one intervention session on one auditory memory would not be sufficient to significantly affect (auditory memories of) all AHs. Therefore, no post-measurements of symptoms of AHs were taken into account. Future research is necessary to examine whether EMDR on emotionally disturbing auditory memories in patients with AHs also has an effect on the AHs themselves (e.g., frequency, severity, or perceived distress).

It was unexpected, but also encouraging, that SUD reductions occurred so quickly during the intervention. However, this resulted in a limitation in the use of analyzable data. Only data from the first round of conditions were used in the analyses, because SUD scores were substantially lowered at the end of the first round, limiting room for (differential) effect during the second round or even resulting in not being able to perform a second round. It is, however, unclear in what way this has affected the results. Another limitation of the study is the sample size. To obtain sufficient power, 36 patients needed to be included in the analyses, but data from only 33 patients could be taken into account. The power was calculated *post hoc* at .79. The power however is a limited issue since Bayesian statistics were used.

In the study, no differentiation was made between subtypes of AHs. All memories of AHs were included as long as the patient could recall an auditory memory of an AHs with sufficient emotional disturbance. McCarthy-Jones et al. (70) suggest that there are many different types of AHs, and they also show the prevalence of the AHs subtype “Nonverbal AHs.” They point out that it seems plausible that the mechanism underpinning such AHs is distinct from the mechanisms underpinning AVHs (70). Also, Woods et al. (71) point out that although AHs are usually understood as predominantly perceptual experiences, nearly half of the participants in their study described their voices either as thought-like or as having both auditory and thought-like qualities. This implies differentiation in AHs. The existence of AHs subtypes suggests that therapeutic interventions may benefit from adaptations according to the subtype. It would be interesting to investigate if different subtypes react to interventions. Treating AHs subtypes, however, may often not be clinically feasible, due to the high number of patients with multiple AHs subtypes. It would however be interesting to look in more detail if there is a difference in effect of dual tasking during recall of auditory memory of different types of AHs and if there is a super-imposed effect of modality-specific taxing. This should be addressed in future research.

In sum, results show that emotionality of AH memories can be reduced by dual tasking and active conditions show stronger effects than the CC. This makes it worthwhile investigating if the use of dual tasking is a possible pathway in treating AHs. Future research should address this and need to include follow up data of the effect on AH symptomatology and the effect on different AH subtypes.

## Ethics Statement

This study was carried out in accordance with the recommendations of the Medical Research Ethics Committee of the University Medical Center Utrecht (The Netherlands).The protocol (NL54140.041.15, protocol number 15/428D) was approved by the Medical Research Ethics Committee of the University Medical Center Utrecht. All subjects gave written informed consent in accordance with the Declaration of Helsinki.

## Author Contributions

SM, LV, and MH designed the research. SM and LV collected the data. SM analyzed the data and wrote the paper. MH and IH critically reviewed the paper. SM, MH, and IH approved the final manuscript.

## Funding

MH is supported by a TOP grant (number: 40-00812-98-12030) from the Netherlands Organization for Health Research and Development (ZonMw). A small grant from the Dutch EMDR association was awarded in June 2015 to SM.

## Conflict of Interest Statement

The authors declare that the research was conducted in the absence of any commercial or financial relatio nships that could be construed as a potential conflict of interest.

## Footnotes

1 Null Hypothesis Significance Testing is added in the appendix.

## Appendix

**Null Hypothesis Significance Testing** Data were analyzed with SPSS (version 23). A repeated-measures within subject ANOVA was conducted, with Condition (VT, AT, and CC) and Time (pre and post) as independent variables and SUD scores as dependent variable. SUD pre scores between conditions did not differ from each other (*F* (2, 64) = 1.08, *p* = .35, η*_P_* ^2^ = .03). There was a significant main effect of Time (*F* (1, 32) = 70.38, *p* < .001, η*_P_* ^2^ = .69), but no significant main effect of Condition (*F* (2, 64) = .05, *p* = .96, η*_P_* ^2^ = .00). Mauchly’s Test of Sphericity indicated that the assumption of sphericity had been violated for the Time x Condition interaction (χ^2^ = 7.05, *p* = .03). Therefore, the Greenhouse–Geisser interaction was used. This showed that the Time x Condition interaction was non-significant (*F* (1.66, 53.19) = 2.53, *p* = 0.10, η_P_^2^ = 0.07). SUD scores, regardless of the condition, decreased from pre to post. Calculating difference scores for all conditions and combining the AT and VT difference scores and comparing these “active” conditions *together* with the CC in a one way ANOVA showed a trend (*F* (1, 32) = 3.46, *p* = .07, η*_p_* = .10).
